# Benefits and Importance of Early Rehabilitation in Patients After Stroke

**DOI:** 10.3390/jfmk11030290

**Published:** 2026-07-25

**Authors:** Harieta Elkova, Galina Mratskova

**Affiliations:** 1 Department of Imaging Diagnostics, Dental Allergology and Physiotherapy, Faculty of Dental Medicine, Medical University of Plovdiv, Plovdiv 4000, Bulgaria; 2 Department of Physical and Rehabilitation Medicine and Sport, Faculty of Medicine, Trakia University, Stara Zagora 6000, Bulgaria; galina.mratzkova@trakia-uni.bg

**Keywords:** stroke, rehabilitation, motor recovery, quality of life

## Abstract

The aim of this article is to provide a literature review of current approaches and recommendations for medical rehabilitation in stroke patients, with the goal of achieving the best possible outcomes for their condition and improving their quality of life. **Materials and Methods**: A narrative review was conducted of articles published in the databases available to us (PubMed, ScienceDirect, Google Scholar, PEDro, ResearchGate) that contain information regarding therapeutic management of stroke and, in particular, interventions aimed at motor recovery and improving quality of life. Included in this literature review were: scientific articles, guidelines on stroke and motor rehabilitation, clinical studies, longitudinal clinical studies, controlled clinical studies, systematic reviews, and meta-analyses published before April 2026, covering a ten-year period. **Results**: Data showing a trend toward higher patient survival rates following acute stroke were identified. For this reason, existing non-pharmacological approaches to motor recovery should be optimized. The rehabilitation process must begin as early as possible. Motor recovery depends on the establishment of motor control and the neuroplastic processes occurring in brain tissue. Rehabilitation is conducted by a rehabilitation team with the patient’s active participation, while monitoring motor rehabilitation potential. **Conclusions**: Rehabilitation is an essential component of post-stroke treatment. When timely and adequate medical rehabilitation is provided to stroke patients, there is a significant improvement in their overall condition and motor functions, as well as in their independence, self-care, and quality of life.

## 1. Introduction

Bulgaria ranks among the highest in the world in terms of stroke incidence and mortality, with a large proportion of stroke survivors left with a high degree of disability and a reduced quality of life. Properly conducted medical rehabilitation is a key component of their treatment. High-quality therapy focused on restoring lost motor function leads to significant improvement in both overall health and in terms of independence, self-care, and quality of life.

Stroke, whether ischemic (cerebral infarction) or hemorrhagic, is a serious condition that leads to significant negative consequences, long-term disability, and permanently impairs patients’ quality of life. According to current data, it ranks second as a cause of death among non-infectious diseases (Global Burden of Disease Study 2021) [1]. Furthermore, stroke plays a leading role as a cause of permanent disability and limited functionality and daily activity [2,3]. Stroke mortality in Europe is expected to decrease by 17% by 2047, while its prevalence is projected to increase by 27% [4]. Based on these projections, it is anticipated that the need for and demand for rehabilitation interventions among stroke survivors will increase [5].

According to data from the National Statistical Institute of Bulgaria, stroke mortality for 2024 is 272.2 per 100,000 (The National Statistical Institute (NSI) Republic of Bulgaria) [6]. Out of the 35,311 cases recorded in 2019, 7175 patients with stroke have died [7]. Diseases of the circulatory system are the most common cause of death. They account for 61% of all deaths in Bulgaria, with one in three fatalities in 2020 caused by “cerebrovascular disease”.

Our World in Data (OWID) statistics [8] on age-standardized stroke mortality show that in 2019, Bulgaria ranked tenth in the world and first among European Union member states. This indicates that the country has a higher mortality rate than nearly 50% of countries with “low-income” economies, and this trend has remained consistent since 1990. In 2010, the mortality rate from stroke was 19.9%, and in 2020 it was 17.6%, with stroke affecting men more frequently. Of all registered cases of stroke in 2020, 42% were fatal. In the remaining cases (28,136), severe disability was observed in about one-tenth of them [9]. This is why the burden of stroke is greater, reaching 2710.4 DALYs per 100,000 people [10]. Stroke survivors have various degrees of disability; in 10% [9] of them, it is severe and requires additional care and assistance from family, relatives, and social services. Treatment of stroke has made significant progress and is implemented in several stages, one of which must include medical rehabilitation.

The European Stroke Organisation (ESO) has developed the European Stroke Action Plan (ESAP) [11], which sets out 30 objectives and 72 research priorities across seven areas in order to improve the healthcare services provided to patients who have suffered a stroke [5]. One of these areas concerns post-stroke rehabilitation, aimed at improving management, outcomes, and quality of life after stroke by 2030 [11]. According to the WHO, the rehabilitation is defined as “a set of measures that help individuals who are experiencing or are likely to experience a disability to achieve and maintain optimal functioning in interaction with their environment” [12]. This definition covers several neurological domains such as motor function, cognitive abilities, and communication; however, no specific principles for conducting motor rehabilitation are presented. A consensus has been reached on the definition of motor rehabilitation following a stroke. The consensus emphasizes that it must include improvements in health, well-being, and quality of life. It also underlines the need to inform the patients that they can experience improvement, even in the long term following a stroke [5].

Medical rehabilitation aims to: restore affected functions to the greatest extent possible, re-train the patient in daily living and occupational skills, and achieve maximum independence in daily life. It can be conducted in hospital, outpatient, and sanatorium settings, under the supervision of qualified medical staff. Treatment and rehabilitation of patients with stroke in most cases is complex and prolonged. Rehabilitation procedures should begin immediately after the acute condition has been managed, even while the patient is in the intensive care unit. It is appropriate to perform breathing exercises and ensure proper positioning of the patient in bed, while medication is administered concurrently with rehabilitation. Given that survival rates after stroke have increased over the past few decades, the quality of life (QOL) of stoke survivors has become a major public health concern [13].

The interaction between social, health, economic, and environmental conditions, that influence human and social development, is what forms the quality of life. It can be defined as subjective well-being, which reflects the difference between individuals’ hopes and expectations and their current experiences. Human adaptation is such that expectations are usually adjusted to remain within the realm of what individuals perceive as possible in life [13].

According to the White Paper of Medical Specialists in Physical and Rehabilitation Medicine (PRM), a PRM specialist serves as a guide for patients with temporary or permanent disability resulting from an injury or illness. The primary goal of PRM is to optimize social participation and improve patients’ quality of life (QOL). This includes helping the patient achieve the highest possible levels of autonomy and independence, including participation in professional, social, and recreational activities, which are an integral part of their human rights [14].

Traditional medical intervention focuses on the disease process, but only as a factor influencing the course of the illness. In contrast, PRM specialists do not separate disease processes from the individual as a whole, nor from their active and passive connections with the surrounding context [15]. Many physicians in Physical and Rehabilitation Medicine (PRM) emphasize this aspect, stating that conventional medicine works against diseases and their consequences, while PRM works toward people’s “functioning” despite diseases and their consequences [16,17].

Physical medicine and rehabilitation specialists provide rehabilitation services in a variety of settings, including acute care hospital wards, specialized rehabilitation centers, and outpatient clinics. The principles of their work are the same regardless of where rehabilitation takes place, although priorities and activities vary according to the treatment needs in those settings. Thus, different rehabilitation and healthcare interventions can be planned to address the varying needs of the patients during the course of their rehabilitation program.

Prolonged discussions concerning the implementation of standardized post stroke rehabilitation protocols have been ongoing within the Association of Physical and Rehabilitation Medicine (FRM) in Bulgaria. In this regard, it is advisable to adopt a national consensus on post stroke rehabilitation harmonized and compliant with internationally endorsed recommendations for the rehabilitation of individuals after stroke.

The aim of this article is to provide a literature review of current approaches and recommendations for medical rehabilitation in stroke patients, with the goal of achieving the best possible outcomes for their condition and improving their quality of life.

## 2. Materials and Methods

A narrative review of scientific literature on post-stroke rehabilitation was conducted. A search was performed in PubMed, ScienceDirect, Google Scholar, PEDro, and ResearchGate, using the following terms: “stroke” OR “cerebrovascular accident” OR “cerebral infarction” AND “rehabilitation” OR “motor recovery” OR “physical therapy” AND “quality of life” OR “daily living activities”. The search was limited to articles published between January 2016 and April 2026. The inclusion criteria covered full-text publications in English and Bulgarian containing data on the application and effectiveness of rehabilitation in stroke patients. In accordance with these criteria, we included clinical guidelines, systematic reviews, meta-analyses, randomized controlled studies, and observational studies. Case reports, editorials, conference abstracts, and articles to which we did not have full-text access, or that did not meet the inclusion criteria, or were duplicate publications, were excluded. The two authors conducted the keyword search independently of one another and, following a discussion, selected the scientific literature according to the established criteria for this narrative review.

## 3. Results

Data showing a trend toward higher patient survival rates following acute stroke were identified. For this reason, existing non-pharmacological approaches to motor recovery should be optimized. The rehabilitation process must begin as early as possible. Motor recovery depends on the establishment of motor control and the neuroplastic processes occurring in brain tissue. Rehabilitation is conducted by a rehabilitation team with the patient’s active participation, while monitoring motor rehabilitation potential.

Preliminary data from the AVERT (Very Early Rehabilitation Trial after stroke) study on immediate physical therapy within the first 24 h indicate good patient tolerance, with no increase in side effects observed [18]. A large proportion of stroke complications (deep vein thrombosis, skin ulcers, contractures, constipation, and hypostatic pneumonia, among others) are associated with immobility. For this reason, early rehabilitation is considered a crucial and fundamental aspect of treatment [19,20].

The ESO Consensus supports early initiation of mobilization as a component of complex rehabilitation, provided that it is individualized and tailored to the patient’s clinical condition [19,20]. In contrast, the AVERT [18] study shows that very early and intensive mobilization does not lead to better functional outcomes and, in patients with severe stroke, may even be associated with a negative effect. Therefore, the contemporary approach is to apply early mobilization in a measured and personalized manner (Table 1).

## 4. Discussion

Stroke recovery is a long-term process that requires consideration of several specific factors, including biological recovery phases, acute medical care, and rehabilitation options. For this reason, we aim to focus on modern approaches and recommendations for medical rehabilitation in stroke patients, enabling the best possible outcomes and improving their quality of life.

### 4.1. Rehabilitation After Stroke

Impairment of motor function in the upper and lower extremities after a stroke requires prolonged rehabilitation, which involves a long-term process of functional recovery. In cases of mild upper limb paresis, up to 80% of patients can be expected to achieve full recovery, whereas in cases of severe paresis, this rate drops to one fifth. It has been found that only about half of stroke survivors who experienced complete paralysis achieve only partial recovery within a six-month period [21]. Recovery of lower limb function is essential for the gait of patients after stroke. Studies show that about 70% of patients who have suffered an acute stroke initially have difficulty walking. After undergoing routine rehabilitation, only half of them is able to move independently [21].

A crucial element of the treatment and rehabilitation of stroke includes the development of an individualized rehabilitation plan that incorporates both the skills and responsibilities of the various team members and the different procedural and structural conditions throughout the various stages of this process [15,22,23]. Contemporary rehabilitation requires an integrative and holistic approach to the patient, as well as an assessment of the effectiveness of the rehabilitation, particularly in patients with socially significant disabling conditions such as stroke, or with reduced health-adjusted QOL [24,25].

The significance of functional assessment must be taken into account, in order to ensure high-quality rehabilitation and improvement in the patients’ independence in daily life and their health-related QOL [26,27]. Unfortunately, high-quality (FTR) rehabilitation for the most common socially significant diseases is complicated by a number of contemporary challenges (scientific, application-related, practical, and organizational) such as low economic standing of the country and its population; increased number of patients, with more of them in advanced stages of disease, and more comorbidities in a single patient.

Rehabilitation, especially for post-stroke patients, is a long-term process that requires the integration of diverse skills from various sectors, as well as the involvement of different organizations and services that extend beyond the healthcare sector. The concept of multidisciplinary rehabilitation is defined by the World Health Organization (WHO) as coordinated provision of multidimensional rehabilitation interventions delivered by various professionals (such as nurses, physical therapists, occupational therapist, social worker, psychologist, and others) associated with various medical specialties (physicians in Physical and Rehabilitation Medicine, neurologists; oncologists, etc.) and aims to influence the patient’s symptoms as well as to maximise functional independence and participation (social integration), using a holistic biopsychosocial model as defined by the ICF [28,29,30].

### 4.2. Neuroplasticity—Influence and Importance for Recovery After Stroke

In relation to neuronal plasticity and structural and/or functional changes in neurons, as well as their interconnections within a network, changes occur in response to these alterations, such as those observed during training or following an injury [31]. The concept of neuroplasticity of the brain cortex defines the modern concept of motor rehabilitation. It has been established that the central nervous system possesses the ability to alter its activity and restructure itself under the influence of external stimulation and training. As a result of this process, new neural connections are formed, new functions are acquired by the preserved brain structures, and lost functions are compensated for by activating alternative pathways in the brain. However, in the cerebral hemisphere affected by the stroke, neuroplasticity is often impaired. This requires the application of specific therapeutic approaches to stimulate it [32,33,34].

The following phases of recovery from stroke have been identified [5]:

- Hyperacute phase: 0 to 24 h after the onset of the stroke;
- Acute phase: 1 to 7 days;
- Early subacute phase: 7 days to 3 months;
- Late subacute phase between 3 and 6 months;
- Chronic phase 6 months or more after the stroke [5].

The so-called “spontaneous neurological recovery,” which involves improvement in function and activities, is actually independent of specific targeted treatment. It may occur within a limited time frame during the first 3 months following the onset of the stroke. It is considered an endogenous recovery process that most likely occurs due to the remaining intact neural structures, which can serve as a basis for reorganization [5,35].

Rehabilitation aimed at restoring motor function is a process that engages stroke survivors to improve their motor function, their activity capacity, and their ability to perform daily activities. For all people with residual motor impairment, no matter how minor it may seem, the aim is to improve their functioning, independence, and participation. Motor rehabilitation should be conducted following regular assessments of mobility and activity using consensus measures, including patient-reported outcomes. Discussing the results with the patient and their caregivers, specific goals are determined to meet the patient’s needs. Targeted motor rehabilitation aims to reduce motor impairments and improve function and performance of activities through training- and usage-dependent mechanisms. The potential for motor and functional recovery varies among patients and across stages of recovery; in the early stages, the behavioral recovery of motor function depends on the primary mechanisms of spontaneous neurological recovery. In the later stages of recovery, additional functional improvements can be achieved by developing compensatory movements [35].

A key component of motor rehabilitation is based on the principles of establishing motor control. Patients are taught to optimize and adapt their mobility, sensory, and cognitive functioning through appropriately dosed, repetitive, goal-oriented, progressive, task- and context-specific rehabilitation. Motor rehabilitation supports people who have had a stroke so that they can maximize their health, well-being, and quality of life [5,36,37,38].

The International Classification of Functioning, Disability, and Health (ICF) defines the interactions between body functions and structures, activities, and individuals’ participation in relation to the environment and personal factors. Body functions may be impaired, and activities and participation in them may be limited. This definition reflects the nature of recovery and distinguishes improvements in earlier and later stages following a stroke [39]. Motor recovery after a stroke most likely occurs as a combination of both spontaneous biological processes and “use-dependent” processes, which include motor learning and the acquisition of lost skills [36]. The interactions between biological processes at molecular, cellular, and physiological levels that occur spontaneously, and how these influence learning mechanisms in the first months following a stroke, have not yet been well studied [36].

Most of the current studies on post-stroke rehabilitation use the term “recovery” as a general term for “change” or “improvement,” without distinguishing between behavioral substitution and motor compensation [40,41].

When the term “motor recovery” is used, it refers to recovery after a stroke, in which the goal is to restore movement or task performance to the level prior to the stroke. This is considered “true recovery” and reflects spontaneous biological recovery processes occurring during the first days and weeks following the stroke. On the other hand, when we use the term “compensation,” it is assumed that the patient performs movements or tasks using non-typical movement patterns. These compensatory movements come at the expense of the quality of the movement or the performance of the tasks using a limb different from the affected one. It is accepted that adaptation and compensation are the result of subsequent motor training processes, which can continue for a very long time, even indefinitely, after a stroke. The results of several studies indicate that, in most patients, spontaneous neurological recovery after a stroke and the restoration of behavioral habits follow an ascending pattern. As a result of this pattern, a plateau is observed within the first 10 weeks following the acute event. This trend is consistent for stroke patients, regardless of their age and/or the type and intensity of the applied therapy [42,43,44]. Spontaneous neurological recovery is observed for the upper limb [45,46] and lower limb [47,48], as well as for somatosensory [49,50] and visual–spatial functions. Furthermore, spontaneous recovery of language abilities has been observed in patients who had aphasia for more than 4 weeks following a stroke [51,52]. Patients who initially had mild to moderate motor impairment typically show early spontaneous neurological recovery of motor function in the upper and lower limbs. Other patients with initially more severe motor impairment may also exhibit early or slightly delayed spontaneous recovery [53,54].

Spontaneous neurological recovery is likely driven by a cascade of various neurochemical reactions triggered by local ischemic brain damage. The improvements caused by the therapy are primarily adaptive and most likely are the result of patients learning to optimize the use of their limbs in order to perform the assigned task. The improvements observed in daily activities essentially reflect the potential for behavioral restoration of impaired bodily functions, which are observed during the first 10 weeks, as well as the training conducted to eliminate residual impairments through the use of compensatory movements [43].

During the motor rehabilitation sessions, various interventions are used to help patients learn to optimize their movements through adaptation and compensation. It is essential that these are applied in the appropriate dosage, performed regularly, goal-oriented, gradually increased in intensity, and specifically tailored to the tasks and training context. The recommendations of the international consensus on stroke recovery clearly identify the need for early assessment of stroke patients’ condition within the first week, followed by assessments every 4 weeks, and at 3 and 6 months post-stroke. These recommendations are based on the understanding that these milestones are associated with important changes in the biological processes underlying stroke recovery [42]. The analysed publications employ diverse therapeutic approaches, methods, procedure durations, and numbers of treatment sessions. This heterogeneity complicates direct comparison across studies and does not allow the formulation of definitive recommendations regarding optimal therapeutic effects.

### 4.3. Assessment Tools for Stroke Patients

The necessity of using standardized assessment methods to determine the condition of stroke patients at a specific point in time is due to the fact that this can ensure transparency in treatment approaches and enable the comparison of outcomes from post-stroke rehabilitation at various levels (regional, national, and global levels). Furthermore, after summarizing the standardized data obtained, an assessment can be made of the effectiveness of the various interventions applied (including information on dosage, timing, method of administration, etc.), which can be useful for clinical practice [55].

The results obtained from motor rehabilitation, together with the results of assessments from other areas of functioning (cognitive, communicative, etc.), must be shared and discussed by patients and the specialists caring for them. In this way, an assessment can be made of the patient’s progress and their expectations as a result of the recovery process. This would facilitate the development of an appropriate individualized rehabilitation plan. Reaching consensus between the patient and the rehabilitation team would ensure the patient’s active participation and cooperation in the rehabilitation process [56].

Making an accurate prognosis based solely on clinical assessment is most difficult in patients with initially moderate to severe motor impairment, even though an accurate prognosis is particularly important for these patients. Very often, prognoses from different physicians may vary, and this could become a reason for unequal access to rehabilitation services [57,58,59]. For this reason, and to minimize the potential for variation, it is recommended to strictly use objective prognostic tools that combine standardized assessment methods. These tools make it possible to predict the likely outcome for each patient, to define rehabilitation goals more precisely, and to tailor the rehabilitation program to the specific patient. Ultimately, this can improve the effectiveness of rehabilitation [56,57].

For people with persistent deficits in movement and mobility, the motor rehabilitation is a key component of stroke treatment [5,60,61]. When developing a rehabilitation program, it is recommended to incorporate various physical methods and occupational therapy, and to assess communication deficits. Although it is desirable to assess cognitive deficits, there is insufficient data regarding recommendations for specific treatment. Patients should be screened for depression during hospitalization, and this monitoring should continue thereafter [29,62].

The significant financial and social consequences of prolonged hospitalization have resulted in increased interest in programs that support patients’ early return to the community [10,63]. For stroke, this problem exists worldwide and is linked to the fact that many studies and scientific literature indicate that the FRM approach can reduce clinical and functional complications and improve recovery and individual outcome quality [3]. In hospitals for acute diseases, and in relation to the financial challenges faced by every National Health System, and for the purpose of reducing costs, rehabilitation interventions are required very quickly, especially for people with stroke. Initially, patients are admitted into the Acute Care Unit, and as soon as possible, they are referred to a specialized rehabilitation unit [64].

A key feature of stroke units is that rehabilitation is carried out by a specialized multidisciplinary team. Significant effectiveness is demonstrated over a 5- and 10-year follow-up period. Rehabilitation conducted under the guidance of a multidisciplinary team to facilitate early discharge, consisting of specialists in the field of stroke, significantly reduces the length of stay for certain patients with mild to moderate impairments [31]. Specialized rehabilitation units are necessary because they are associated with increased survival and improved functional outcomes for patients receiving treatment there [63,65].

The outcome of rehabilitating these conditions depends on the severity and complexity of the neurological impairment and its impact on the individual’s activity and participation, as well as on a wide range of professional skills. The main components of a properly structured and individualized rehabilitation program should include: medication, periodical courses of physical therapy and rehabilitation, and a hygiene and dietary regimen [29]. It must be emphasized that the patient’s active participation in the rehabilitation process is a necessary and mandatory condition for ensuring a positive outcome from rehabilitation. Rehabilitation is a long-term and coordinated process that should be aimed not only at survival but also at achieving better health and participation, and thereby a better QoL for the stroke patient [66].

Early rehabilitation covers the period from the onset of the condition until the patient is discharged from the hospital. It is an important component of treatment and should begin as soon as the patient is hemodynamically stable [30,67]. It is recommended to begin rehabilitation upon admission to the acute stroke unit, and it is advisable to continue it throughout the first year following the stroke. The duration and intensity are gradually increased, but this must be strictly assessed on an individual basis by a physical and rehabilitation medicine specialist. The methods used in rehabilitation are designed so that their application is effective in the functional recovery of organs and systems, as well as in the development of the body’s compensatory mechanisms, enabling the patient to subsequently perform work activities and/or self-care. Compared to late rehabilitation, early rehabilitation improves clinical efficacy and can significantly enhance self-care abilities, activities of daily living, and neurological functions in patients with ischemic stroke [68]. Although the available data support the benefits of rehabilitation, the limited number of randomized controlled trials conducted specifically in stroke patients does not allow for high-level recommendations to be formulated.

### 4.4. Access to Rehabilitation

Access to rehabilitation for patients with stroke is difficult in many locations and is shaping up to be a global problem. It is expected to become increasingly significant, particularly in countries with a large geographic area and/or a high proportion of rural areas, due to a shortage of rehabilitation staff or limited access. The lack of such care services and/or barriers to accessing them leads to increased stress and anxiety and affects patients’ quality of life [23,69].

Access to early rehabilitation in Bulgaria, on the other hand, depends directly on the availability of a hospital department with physicians trained in physical and rehabilitation medicine, as well as qualified rehabilitation specialists, physical therapists, and other specialists. Early rehabilitation begins in neurology wards at hospitals equipped with a physical and rehabilitation medicine department. A mobile rehabilitation team is formed which visits the patient on-site to provide rehabilitation; after discharge from the neurology department, the stroke patient has the option to continue with inpatient rehabilitation at a physical and rehabilitation medicine facility. Essentially, this opportunity for early rehabilitation has two limitations. On one hand, there are high-level hospitals that have departments covering nearly all medical specialties and a large number of specialists and equipment (including modern technologies for robot-assisted rehabilitation, etc.), on the other hand, there are hospitals that do not have FRM departments and lack such specialists and specialized staff. In the latter case, patients’ access to medical care funded by the National Health Insurance Fund/NHIF/is limited.

The second limiting factor is the duration of the rehabilitation provided to every person with health insurance in Bulgaria. According to the rules established by the National Health Insurance Fund, every insured patient who has suffered a stroke is entitled to two rehabilitation courses per year (the second course is provided only after a proven deterioration in the patient’s motor function), and the costs of these courses are covered by the health insurance. As of today, the duration of inpatient rehabilitation financed by the NHIF is seven days. Of course, there is no limitation on the rehabilitation duration course, but since it is not covered by the NHIF, it becomes a problem—health-related and financial—for both the stroke patients and the physical therapy specialists, given that recovery from a stroke is a process that takes time to achieve independence in daily life and improve functional ability.

The provision of outpatient rehabilitation in Bulgaria is insufficiently regulated with respect to its organisation, accountability, and implementation, which in turn leads to blurred responsibilities. As a result, stroke patients frequently pay for outpatient rehabilitation services without being aware of the quality or the appropriate scope of the care they receive.

The general practitioner plays a key role in organizing and overseeing the rehabilitation process. He or she must assess and refer stroke patients for rehabilitation, but this is often subject to restrictions and limits set by the NHIF, which hinders patients’ access to treatment.

The existing organizational, managerial, financial, and economic problems in Bulgaria’s public healthcare system significantly impact the possibilities for effective rehabilitation of stroke survivors, thereby increasing the burden of this condition on patients, their families, and society as a whole, and negatively affecting their quality of life.

Modern rehabilitation is increasingly being used in the treatment of stroke patients, as evidence of its benefits continues to accumulate. In addition to addressing motor deficits, there is also compelling evidence of improved quality of life for patients, which is of essential importance to them [68,70,71]. The success of rehabilitation in stroke survivors depends on the severity and complexity of the neurological impairment and its impact on the individual’s activity and participation, but also on a wide range of professional skills. Rehabilitation is a long-term and coordinated process that must be directed toward the ultimate goal—first and foremost survival, but also, obviously, achievement of a better level of health and participation, and a better quality of life [66].

### 4.5. Key Findings for Clinical Practice

The present literature review shows that post-stroke rehabilitation can be a valuable component of the complex treatment of stroke survivors. During the rehabilitation process, certain recommendations must be followed to improve clinical outcomes:

- Initiate rehabilitation as early as possible, within 24–48 h of stroke onset, once hemodynamic stability has been achieved;
- Standardized tools (e.g., the National Institutes of Health Stroke Scale (NIHSS), the Functional Independence Measure (FIM), the Barthel Index, etc.) should be used to assess rehabilitation potential;
- Assessment of rehabilitation potential should be performed regularly: upon admission, after 1 week, 1 month, 3 months, and 6 months;
- Specialized therapy sessions must be conducted daily (at least five days a week) for a minimum of 45 min by qualified therapists;
- The rehabilitation team caring for a patient after a stroke must include: a physician specializing in physical and rehabilitation medicine, a physical therapist, an occupational therapist, a logopedist, a psychologist, and a social worker;
- Patients with moderate and severe disabilities should be promptly referred to specialized inpatient rehabilitation facilities;
- Screening for symptoms of depression, cognitive impairments, and caregiver burden should be conducted during the recovery period.
- Guidelines for Managing the Post-Stroke Rehabilitation Process in Bulgaria:
- It is necessary to develop and implement clinical protocols for stroke patients that include specific interventions, an algorithm for their implementation, and a system for evaluating the quality of the rehabilitation provided;
- Periodic assessment of motor function and quality of life after a stroke is necessary to determine the need for longer-term rehabilitation and subsequent courses of treatment;
- The introduction of mandatory questionnaires or monitoring scales for stroke patients’ treatment in rehabilitation departments nationwide.

### 4.6. Limitations

This review has several limitations. First, as a narrative review, it does not include a systematic search or a quantitative meta-analysis. Second, the search was limited to full-text articles in English and Bulgarian, which potentially excludes relevant studies published in other languages and/or articles whose full text was not available to us. On the other hand, the emphasis on Bulgarian data may limit its generalizability to other regions. It cannot be ruled out that the authors may have exhibited some bias in the selection and interpretation of the articles included in this review. In addressing the issue of the prolonged debate and the delayed formulation of a national consensus on the rehabilitation of stroke patients, the authors have relied largely on published guidelines rather than on high-quality primary evidence. This is probably the reason why greater attention is paid to the guidelines already issued by authoritative international institutions, which can serve as a basis for developing a national program to achieve consensus.

## 5. Conclusions

Rehabilitation is an essential component of post-stroke treatment. When timely and adequate medical rehabilitation is provided to stroke patients, there is a significant improvement in both their overall condition and motor functions, as well as in their independence, self-care, and quality of life. The rehabilitation process depends on the correct assessment of motor function, the degree of its impairment, and the monitoring of its changes. Motor recovery is carried out by a team of medical specialists, who develop an individualized rehabilitation program tailored to the patient’s needs. Addressing impairments in motor function is a prerequisite for improving daily activities and quality of life after a stroke. Future high-quality clinical trials assessing the effectiveness of post-stroke rehabilitation are needed.

## Figures and Tables

**Table 1:** Comparison between the ESO recommendations and the results of the AVERT study regarding early mobilization after an ischemic stroke.

Indicator	ESO Consensus [9,11]	AVERT Study [18,19,20]
Main Recommendation	Recommends early mobilization as part of the standard of care.	No significant improvement in functional outcome with very early mobilization was observed.
Start of Mobilization	24–48 h after the stroke, in stable patients.	Within the first 24 h, often with higher intensity and frequency.
Intensity	Individualized, gradually increasing depending on the patient’s condition.	More intensive and more frequent mobilization compared to standard care.
Effect on Functional Recovery	Expected benefit for the recovery and prevention of complications with careful patient selection	No statistically significant improvement in functional outcomes was observed at 3 months.
Risks	Emphasizes the need for individualized assessment and monitoring.	Evidence of potential harm in certain subgroups, particularly in patients with severe stroke.
Clinical significance	Early rehabilitation, but with a personalized approach is promoted.	The study warns that mobilization that begins too early and is too intense is not appropriate for all patients.

## Data Availability

The raw data supporting the conclusions of this article will be made available by the authors on request.
